# Can pocket parks be compared to community parks in the restoration effect of physical and mental health for young adults? A comparative experiment in high-density urban Green spaces

**DOI:** 10.3389/fpubh.2025.1610497

**Published:** 2025-06-02

**Authors:** Yuzhe Zhang, Yunhong Hu, Yunqi Wei, Yuge Xie

**Affiliations:** ^1^College of Landscape Architecture, Nanjing Forestry University, Nanjing, China; ^2^College of Landscape Architecture, Beijing Forestry University, Beijing, China

**Keywords:** pocket park, community park, public health, restoration effect, green space, urban forest

## Abstract

**Introduction:**

Urban intensification limits the availability of green space, leading to the rise of pocket parks as a strategy approach to urban greening. Unlike conventional community parks, pocket parks are smaller in scale and function, necessitating further investigation into their capacity to support psychophysiological restoration. Specifically, understanding which landscape elements within pocket parks most effectively facilitate recovery in young adults is essential to determining whether these spaces can achieve restorative outcomes comparable to those of larger community parks.

**Methods:**

This study examines the restorative efficacy of pocket parks by comparing two pocket parks and two community parks in Nanjing, each representing distinct typologies—one prioritizing vegetation and the other prioritizing artificial environments, including hardscape activity zones. Eighty participants (aged 18–28) were randomly assigned to different park types, where their psychological and physiological responses were assessed using standardized surveys and biometric measurements.

**Results:**

The findings indicate that, despite their smaller size, well-designed pocket parks with diverse landscape features significantly enhance users’ psychological relaxation and emotional well-being, achieving restorative effects comparable to those of community parks. Notably, this research highlights the critical role of hardscape activity areas in promoting restoration among young adults, an overlooked aspect of park design.

**Discussion:**

These results underscore the imperative of prioritizing “quality” in park design and renovations, advocating for integrating diverse landscape elements within limited spaces to optimize holistic recovery in urban environments.

## 1 Introduction

The accelerating pace of urbanization has precipitated a global crisis in mental and physical health, particularly among young adults residing in high-density cities (1). Chronic exposure to environmental stressors such as noise pollution, population density, and reduced access to green spaces has been associated with high rates of anxiety disorders, depressive symptoms, and cardiovascular ailments (2, 3). Additionally, prolonged sedentary behavior in office settings, characterized by minimal physical activity, further elevates the risk of developing these health issues (4). Urban green spaces (UGS) have increasingly gained recognition as vital interventions, offering restorative environments that mitigate stress (5, 6), enhance cognitive function (7, 8), decrease depression risk (9, 10), and promote physical activity beneficial to immunity and cardiovascular health (11, 12). Existing theoretical frameworks, such as Ulrich’s Stress Reduction Theory (SRT) and Kaplan’s Attention Restoration Theory (ART), suggest that natural environments facilitate psychological recovery through mechanisms like fascination, compatibility, and escape (13, 14). Pocket parks, characterized by their compact size (<1 hectare), are strategically placed within densely built environments and typically feature simplified vegetation, which might differ significantly in their restorative effectiveness (15). For young adults who are constrained by limited time, the accessibility and immediacy of pocket parks may compensate for their smaller scale. Nonetheless, empirical evidence comparing their efficacy to larger community parks remains scarce.

Previous studies have explored how both pocket parks and community parks contribute to physical and mental health recovery (15–17). Findings suggest that both types of parks positively affect mental fatigue reduction and emotional well-being improvement, as demonstrated by various physiological and psychological indicators. Research has further shown that greater availability of neighborhood green spaces strongly correlated with reduced symptoms of depression, anxiety, and stress (18, 19). Community parks additionally foster social interaction, offering amenities like fitness equipment, walking paths, and sports fields, thereby enhancing communication and community cohesion (17, 20). These activities significantly bolster urban residents’ mental health recovery. However, the construction of large community parks faces substantial limitations in high-density urban areas due to scarce land resources. The contradiction between land availability and the desire to establish community parks is increasingly apparent (21). Pocket parks, conversely, enable the expansion of urban green networks into neighborhoods where access to traditional parks is constrained (22–24). Therefore, it is essential not only to investigate the impact of pocket parks and community green spaces on the physical and mental health of residents living in densely populated urban areas but also to examine the differences in restorative effects between the two types of urban parks.

Recent studies on the environmental attributes of pocket parks and community parks have primarily focused on two key domains: the biophysical environment, which includes elements such as flora and aquatic, and the built environment, including hardscape activity zones and recreational fitness infrastructure (25, 26). While existing literature primarily highlights the restorative benefits of vegetation; some studies suggest that urban amenities may also have the same effects (27). Examining the interplay between these elements is crucial, as integrating diverse vegetation with well-designed urban amenities may enhance the overall user experience. Research indicates that landscape features, such as floral communities and water features, as well as fitness trails and equipment, positively influence park visitors’ stress reduction and relaxation (28). Studies also suggest that a rich vegetation hierarchy significantly enhances the perceived restorative effect in community parks, whereas basic amenities have a relatively minor impact (25, 29). Research on pocket parks indicated that both vegetation and recreational facilities play a key role in promoting mental and physical health (30). Moreover, relaxation and exercise facilities not only enhance restorative effects but also influence psychological characteristics. Therefore, further investigation into the interaction between environmental determinants and activity spaces is merited.

Prior research indicates that pocket parks offer restorative benefits for residents’ mental and physical well-being. Nevertheless, a comparative analysis of the restorative potential of pocket parks versus larger community parks remains understudied. Therefore, it is imperative to investigate whether pocket parks could offer comparable restorative benefits to those provided by larger community parks. Further research is also needed to identify the specific park attributes that most effectively enhance their restorative potential. This study will focus on whether the addition of specific landscape elements can enhance the restorative effects of pocket parks to a level comparable to that of community parks. Furthermore, it will investigate which elements of pocket parks and community parks have a more significant restorative impact on young adults.

This study aims to compare the restorative effects of pocket parks and community parks in high-density urban areas and examine whether pocket parks can achieve restorative benefits comparable to those of community green spaces. Additionally, it seeks to identify the key factors that enhance the restorative effects of small-scale parks, so as to provide insights for the development of an interconnected park network in high-density urban areas. The ultimate goal is to support the creation of high-quality micro-green spaces that contribute to residents’ well-being. To achieve this, we evaluate the restorative effects of pocket parks versus community parks on the mental and physical health of young adults (aged 18–28 years) in a high-density urban environment through a controlled, comparative experiment. A mixed-methods approach is employed, integrating physiological data (e.g., blood pressure, pulse, heart rate variability, and blood oxygen saturation) with standardized psychological scales (e.g., PRS, PANAS, and POMS) to test the following hypotheses: (1) Despite spatial constraints, pocket parks have the potential to achieve comparable restorative effects to community parks; (2) Vegetation richness has a stronger moderating effect on the restorative efficacy of pocket parks; and (3) constructed activity spaces and facilities play a more critical role in determining restorative outcomes.

## 2 Materials and methods

### 2.1 Experimental sites

**Figure 1:**
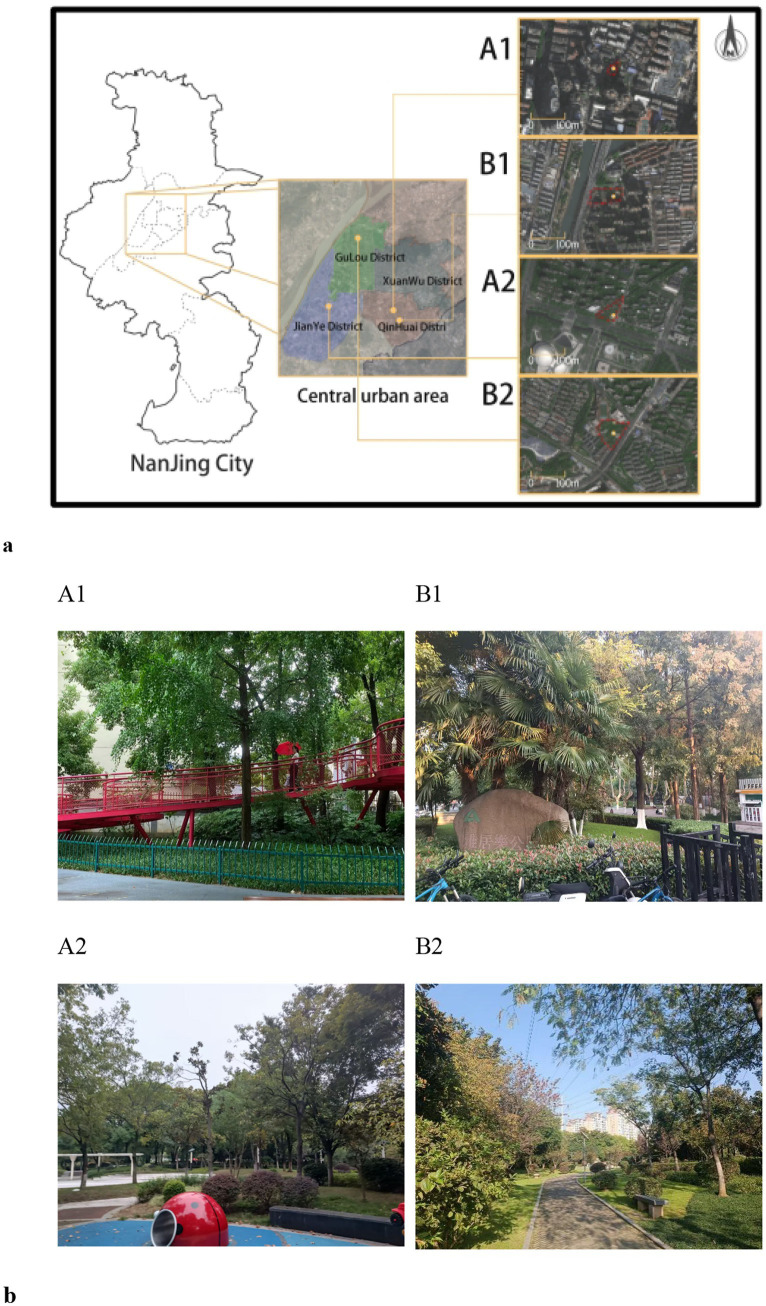
The experiment sits. **(a)** The map of experimental locations (**A1**: Huowa Lane Pocket Park; **A2**: Cangshanlu Pocket Park; **B1**: Yajule Park; **B2**: Yingyan Park). **(b)** Photos of four experiment sits.

Two groups of pocket parks (Group A1, Group A2) and community parks (Group B1, Group B2) with different levels of green space were used as sites for this study (Figure 1). One pocket park and one community park featured a higher proportion of greenery, dominated by planting space, while the other two prioritized activity space, maintaining a green coverage rate of over 65%. A comparative experiment was conducted simultaneously. The classification of pocket parks and community parks follows the latest urban green space standard (CJJ/T85-2017). “Pocket parks are defined as having independent sites, small scale or diverse forms, convenient for residents to use, with certain recreational functions. They are areas of less than 1 hectare and a green coverage rate of not less than 65%. Community parks are green spaces with independent land use and basic recreational and service facilities, mainly serving residents within a certain community area to carry out daily activities. Their scale should be more than 1 hectare.”

### 2.2 Participants

Before recruitment, a power analysis was conducted using G*Power (version 3.1.9.7) to ensure an adequate sample size for detecting the expected effect. A paired-sample t-test (two-tailed) with an effect size (Cohen’s d) of 0.5, a significance level (*α*) of 0.05, and a power (1-*β*) of 0.9 determined a minimum sample size of 38 (19 per group) (29). Ultimately, 80 participants were recruited, evenly divided into four groups of 20.

Rising social competition and academic pressure have heightened stress among young adults, particularly those entering the workforce or preparing for higher education and career exams (2). Therefore, this study focused on young adults aged 18–28, primarily students, with a few employed participants. Questionnaires and interviews confirmed that participants were experiencing stress related to education and employment (30). Recruitment was led by the School of Landscape Architecture, Nanjing Forestry University, targeting freshmen to third-year graduate students. A small number of young adults with a maximum work experience of 2 years. Participants were evenly distributed by gender and age: 41 females and 39 males. Group A1 included 11 males and 9 females, Group A2 had 10 males and 10 females, Group B1 consisted of 8 males and 12 females, and Group B2 had 10 males and 10 females. Students from Nanjing Forestry University traveled by bus, while external participants were grouped by residential proximity.

All participants met normal physical and psychological criteria, with no psychiatric disorders (e.g., depression, schizophrenia, and dysphoria) or sensory/cognitive impairments. They received full briefings before providing informed consent. This study was conducted in accordance with the Ethics Committee of Nanjing Forestry University.

### 2.3 Procedure

Eighty participants were randomly assigned to four parks, with 20 per scenario. Experiments were conducted on sunny days with optimal conditions—moderate temperatures (19–24°C) and mild winds (2–3 m/s on the Beaufort scale)—between 13:00 and 15:00. Testing took place on November 8 and 9, 2024: Pocket Parks Group A1 and Community Parks Group B1 on November 8, and the remaining groups on November 9. Both groups tested each day followed the same schedule. On experiment days, conditions included air pressure of 1,021 hPa, wind speed of 2.18 m/s (northeast), humidity at 73.4% RH, and air temperature of 21 ± 3°C. Recorded noise levels were: A1: 61 ± 5 dB, A2: 57 ± 3 dB, B1: 62 ± 4 dB, and B2: 60 ± 3 dB. Environmental data were collected using the YuWen YEM-70 L precision temperature and humidity meter, an atmospheric pressure recorder, and a Laiwu handheld anemometer. Audio levels were measured with an Apple Watch Series 9. All four experimental days had similar weather conditions. Participants’ physiological and psychological states were assessed before and after their park visits.

**Figure 2:**
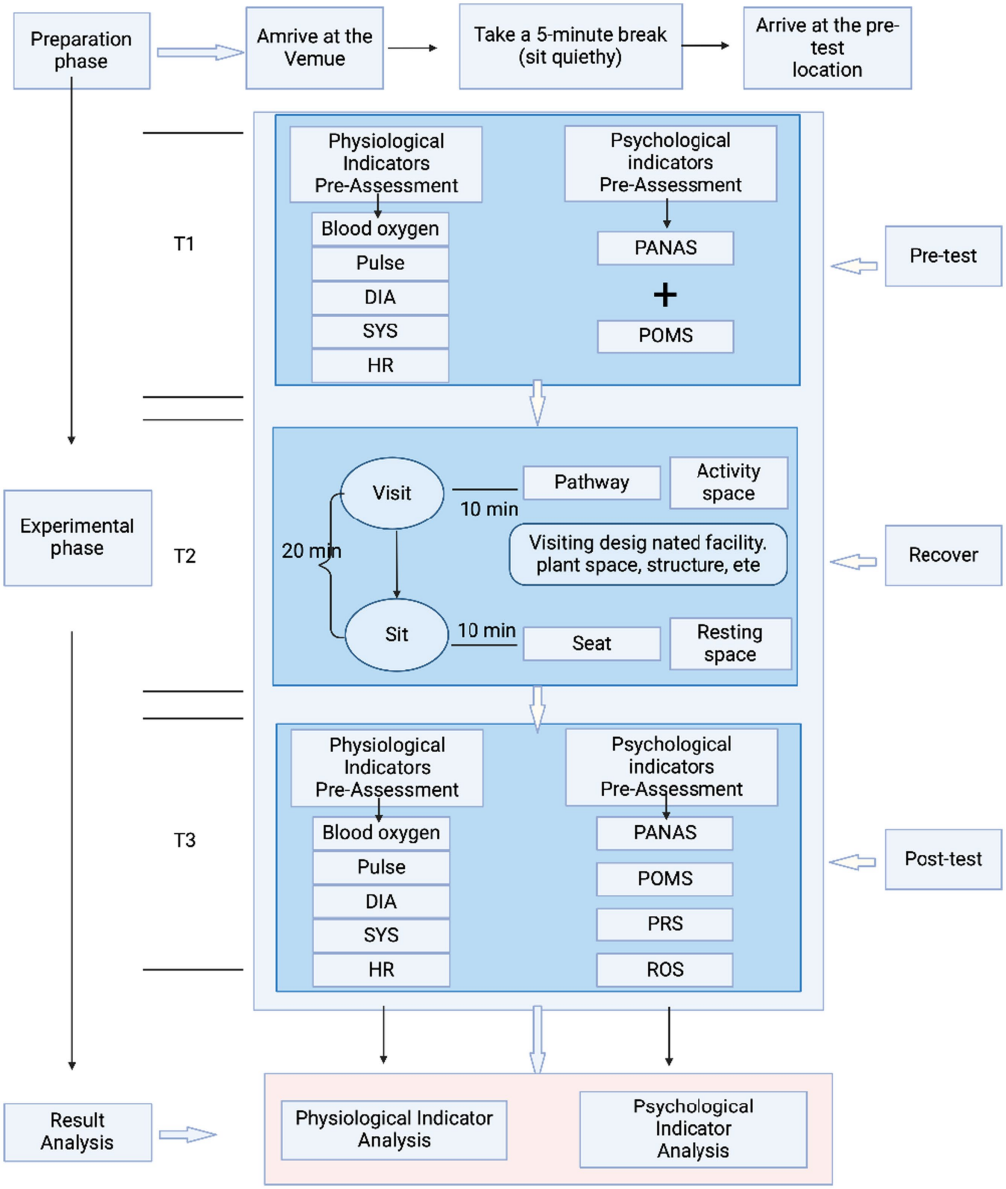
Flowchart of the experimental procedure.

Participants completed the PANAS and POMS questionnaires (pre-test) 2 h before their park visits. Upon arrival, they wore a wrist blood pressure monitor (Omron HEM-6221) and a portable Holter monitor (Lepu ER1). After a 5-min rest at a designated site, initial blood pressure, oximetry, and pulse measurements were taken (T1). During the recovery phase (T2), participants walked for 10 min along a controlled route at a maximum speed of 4.5 km/h. They then either rested quietly or used designated recreational facilities, avoiding vigorous activity. To prevent crowding, participants were divided into five groups, each accompanied by an assistant. After the 20-min session, a second round of oximetry, blood pressure, and pulse measurements was taken. Research indicates that a 20.5-min park visit yields the highest overall accuracy in predicting life satisfaction, suggesting that park design should encourage visitors to spend a minimum of 20 min within the space. Consequently, this experiment mandates a 20-min duration for participants’ park visits to optimize restorative effects (31). Participants were prohibited from using mobile phones, conversing, eating, drinking, or smoking during the visit. Post-test assessments were conducted in park pavilions or shaded areas, where participants completed the PANAS, POMS, and Park Recovery Effectiveness Assessment Questionnaire, including PRS and ROS scales, to capture their genuine responses. The experimental procedure is illustrated in Figure 2. To ensure internal validity and control confounding variables (e.g., physiological responses influenced by unpredictable social behaviors), participants were instructed to avoid interactions during park visits. While this design choice may limit ecological validity, it allowed precise measurement of environmental effects on individual recovery. Future studies will incorporate free social interaction scenarios to compare outcomes.

### 2.4 Measurements

#### 2.4.1 Physiological and psychological indices measurements

Blood pressure parameters assess cardiovascular function, reflecting heart efficiency and vascular health. Studies show that walking in forests and suburban areas effectively lowers blood pressure (32), highlighting the natural environment’s role in cardiovascular regulation. Heart rate, a key indicator of autonomic nerve activity, along with blood pressure, is widely used in outdoor health studies due to its sensitivity, quick measurement, low error rate, and minimal environmental limitations. These indices are essential for evaluating the natural environment’s impact on individual health.

Four psychological questionnaires were employed to evaluate the participants’ responses to the examined environments in this research: Positive and Negative Affect Schedule (PANAS), Profile of Mood States (POMS), Perceived Restoration Scale (PRS), and Restoration Outcomes Scale (ROS). The Positive and Negative Affect Schedule (PANAS) was created by Watson, Clark, and Tellegen in 1988 (33). PANAS and POMS use a 5-point Likert scale (1 = very slightly/not at all, 5 = extreme). The POMS scale includes 30 adjectives measuring six emotional states: Tension, Anger, Depression, Vigor, Fatigue, and Confusion. A positive emotional state is reflected by low negative affect and high vigor scores, with established reliability and validity. The PRS employs a 22-item Chinese version (Cronbach’s alpha: 0.80–0.91) (34), widely used in previous research. It consists of four components: ‘Being Away’ (5 items), ‘Fascination’ (6 items), ‘Extent’ (5 items), and ‘Compatibility’ (6 items), rated on a 7-point scale (1 = not at all, 7 = completely). The ROS uses a 7-point Likert scale to assess six recovery outcomes, including relaxation, vitality, focus, and worry alleviation. Extensively used in studies on natural environment restoration, it has demonstrated strong validity and reliability (35).

#### 2.4.2 Assessment of environmental quality indicators

Research indicates that natural elements, such as flora and greenery, significantly enhance physical and mental health. However, artificial elements, including activity spaces and recreational facilities, also contribute to restoration. Additionally, external environmental factors and site hygiene influence recovery effectiveness. Since none of the selected parks contained water bodies, this study assessed restoration effects using plant species, colors, trees, shrubs, and lawns—elements proven to have restorative benefits (29, 36). For artificial elements, hard spaces were examined, including activity areas, private spaces, recreational facilities, and rest zones, with a focus on seating quality and orientation (37, 38). External surroundings and cleanliness were also considered, as prior research highlights their importance (30). A questionnaire was conducted (Table 1) to establish a subjective evaluation system integrating natural and artificial factors, using a 5-point Likert scale. Reliability and validity testing confirmed strong consistency, with a Cronbach’s coefficient of 0.922, a standardized item Cronbach’s Alpha of 0.925, and a KMO measure of 0.899. This questionnaire aims to determine whether natural or artificial elements play a greater role in influencing psychological and physiological restoration, helping pocket parks achieve restorative effects comparable to community parks.

**Table 1:** A subjective evaluation system for various park indicators, including the specific park metrics, explanations, and associated questions.

Factors	Classification	No.	Indicators	Interpretation	Question
Natural	plant	1.	Plant species	The diversity of plant species is obvious within the park.	The variety of plant species here is abundant, providing me with a sense of pleasure.
		2.	Plant color	The diversity of plant species found within the park.	The variety of colorful plants here is truly pleasing to the eye.
		3.	Richness of trees and shrubs	Evaluate trees and shrubs in the park.	The lush trees and shrubs here provide me with a sense of comfort.
		4.	Lawn	Evaluate the lawn in the park.	The lawn here makes me feel relaxed.
Artificial	Landscape structures	5.	Landscape structures (pavilions, landscape bridges, landscape pergolas, etc.)	The landscape structures of the park, such as pavilions, landscape bridges, landscape pergolas, etc.	The landscape bridges, pavilions, and other structures here uplift my spirits.
	Spaces	6.	Activity Space	The areas provided by people in the park include all kinds of leisure, entertainment, sports, and social activities.	The activity space here can alleviate my stress and fatigue.
		7.	Private Space	Provide relative isolation and quiet areas to individuals or small groups in the park.	The well-designed private space here makes me feel comfortable.
	Activity facilities	8.	Entertainment and fitness facilities	The facility provides people with entertainment and workouts.	The diverse range of recreational facilities here brings me great joy.
	Recreational facilities	9.	Seat Comfort Level	Whether the seat can provide a comfortable experience.	The seating comfort here is very high.
		10.	The orientation of the seating	Whether the seat is convenient to watch the scenery.	Sitting here makes me feel comfortable, allowing me to enjoy the scenery.
	Other factors	11.	External Environment of the Venue	The impact of the off-site environment on this park.	The external environment of the venue has a minimal impact on me and will not affect my psychological relaxation.
		12.	Environmental sanitation conditions	The environmental sanitation of the park.	The hygiene standards in this region are satisfactory.

Through the collection of online resources combined with on-site investigations and measurements, the following indicators were derived for the park’s environmental elements. A field investigation was employed to survey the number of facilities within the park, and the XinSe APP was utilized to identify and estimate the number of plant species, resulting in the following data (Table 2). The coverage rates of trees, shrubs, and lawns are calculated by dividing the area covered by trees, shrubs, and lawns by the total area, which includes overlapping sections.

**Table 2:** Measurement results of various indicators within the parks.

	Indicators about park elements	Pocket park I (Group A1)	Pocket park II (Group A2)	Community park I (Group B1)	Community park II (Group B2)
Basic properties of the site	Site Area	1821 m^2^	9,402 m^2^	14,251 m^2^	17,450 m^2^
	Green Space Rate	67%	82%	70%	77%
	Hard Ground Rate	33%	18%	28%	23%
Natural Features	Plant species (approx.)	18	39	42	55
	Tree coverage rate	42%	32%	36%	38%
	Shrub coverage rate	38%	42%	24%	34%
	Lawn coverage rate	21%	33%	41%	29%
Event spaces	Number of hard event spaces	1	3	3	2
	Hard event space area	825 m^2^	2,608 m^2^	4,275 m^2^	4,013 m^2^
Facility	Number of recreational facilities	0	7	12	0
	Number of rest seats	6	10	24	9
	Number of landscape structures	1	2	4	1
	Number of rest pavilions	1	2	3	1

This study employs both subjective and objective evaluations of the landscape environmental elements within the selected pocket parks and community parks. The subjective assessment is conducted through a questionnaire, allowing participants to assign scores, while the objective evaluation involves on-site measurements to quantify the proportion of these elements within the parks.

#### 2.4.3 Data analysis

All statistical analyses were performed using SPSS 27.0. For physiological data, we conducted means and multifactorial variance analyses of pre- and post-test measurements to evaluate recovery effects among groups. Variance analyses were also applied to PANAS and POMS scale data, both within and between groups. The PRS scale assessed inter-group differences across dimensions, while the ROS scale calculated total scores for recovery effect comparisons. A correlational regression analysis was then executed between total recovery effects from the ROS and the park element assessment questionnaire. To explore the relationship between physiological recovery differences and park environmental elements, regression analyses were utilized to examine the variations in physiological indicators alongside field-measured environmental factors.

## 3 Results

### 3.1 Physiological indices

#### 3.1.1 Results of the analysis of variance

In this experiment, five physiological indices were measured: systolic blood pressure (SYS), diastolic blood pressure (DIA), pulse, heart rate, and blood pressure oximetry. The findings from the two-way mixed-model ANOVAs are displayed in Table 3. Physiological indicators were significant between groups for oxygen (*F* = 3.348, *p* < 0.05), pulse (*F* = 5.249, *p* < 0.01), diastolic blood pressure (*F* = 4.451, p < 0.05), and heart rate (*F* = 3.122, *p* < 0.05). These levels of significance indicated that the different types of parks differed in the degree of physiological recovery and contributed significantly to the changes in these physiological parameters, whereas systolic blood pressure (*F* = 2.266, *p* > 0.05) was not significantly different. Regarding the temporal aspect, the differences observed in pre- and post-measurements were statistically significant for pulse (*F* = 6.976, *p* < 0.01) and heart rate (*F* = 7.051, *p* < 0.001), while no significant differences were found for oximetry, diastolic blood pressure, and systolic blood pressure. The interaction between group and time had no significant effect on blood oxygen (*F* = 1.415, *p* > 0.05), pulse (*F* = 0.096, *p* > 0.05), diastolic pressure (*F* = 0.224, *p* > 0.05), systolic pressure (*F* = 0.141, *p* > 0.05), and heart rate (*F* = 0.238, *p* > 0.05). This indicated that the effects of group and time on these physiological parameters were independent of each other, with no significant interaction effect observed (Table 3).

**Table 3:** Results of multivariate analysis of variance (ANOVA) for physiological variables, degrees of freedom, and F statistics.

	DF	SpO_2_	Pulse	DIA	SYS	HR
Site	3	3.348*	5.249**	4.451*	2.266	3.122*
Time	1	2.589	6.976**	3.865	1.167	7.051***
Site* Time	3	1.415	0.096	0.224	0.141	0.238

*p < 0.05; **p < 0.01; ***p < 0.001; DIA, diastolic blood pressure; SYS, systolic blood pressure.

#### 3.1.2 Results of the physiological indices

The outcomes of the *post-hoc* analysis performed subsequent to the ANOVA were presented in Table 3, where means were denoted with lowercase letters to indicate that they differed from means represented by distinct letters. All four groups of parks showed a positive trend in physiological indicators, with a slight increase in blood oxygen indicators and a decrease in all other indicators. This indicated that people recovered in all physiological indicators after a short exposure to nature, which was consistent with previous studies. However, among the differences in recovery results, the change in pulse rate was more pronounced in community parks than in pocket parks, with little difference in other indicators (Table 4).

**Table 4:** Means and SD of physiological measures in four sites during the experiment.

Group	Time	SpO_2_		Pulse		DIA		SYS		HR	
		Mean	SD	Mean	SD	Mean	SD	Mean	SD	Mean	SD
Pocket Park (A1) With activity space	pre	98	0.86	80.95	9.61	81.25	16.76	118.85	22.47	82.90	13.18
	post	98.4	0.5	77.55	10.88	75.9	12.46	118.3	15.05	75.80	12.28
Pocket Park (A2) With rich plant	pre	97.3	1.42	92.7	16.6	68.95	14.99	109.75	21.62	90.85	16.11
	post	98	1.08	87.5	15.61	67.8	11.58	107.8	17.12	86.20	13.91
Community Park (B1) With activity space	pre	98.2	0.52	88.4	11.54	77.3	14.56	117.15	17.83	86.35	12.03
	post	98.2	0.52	82.45	11.71	72.75	10.76	112.25	11.27	83.10	12.69
Community Park (B2) With rich plant	pre	98.3	0.80	87.65	10.14	77.95	8.82	114.75	12.7	88.65	15.15
	post	98.2	1.54	81.7	10.09	73	11.14	110.4	16.38	80.95	12.21

**Figure 3:**
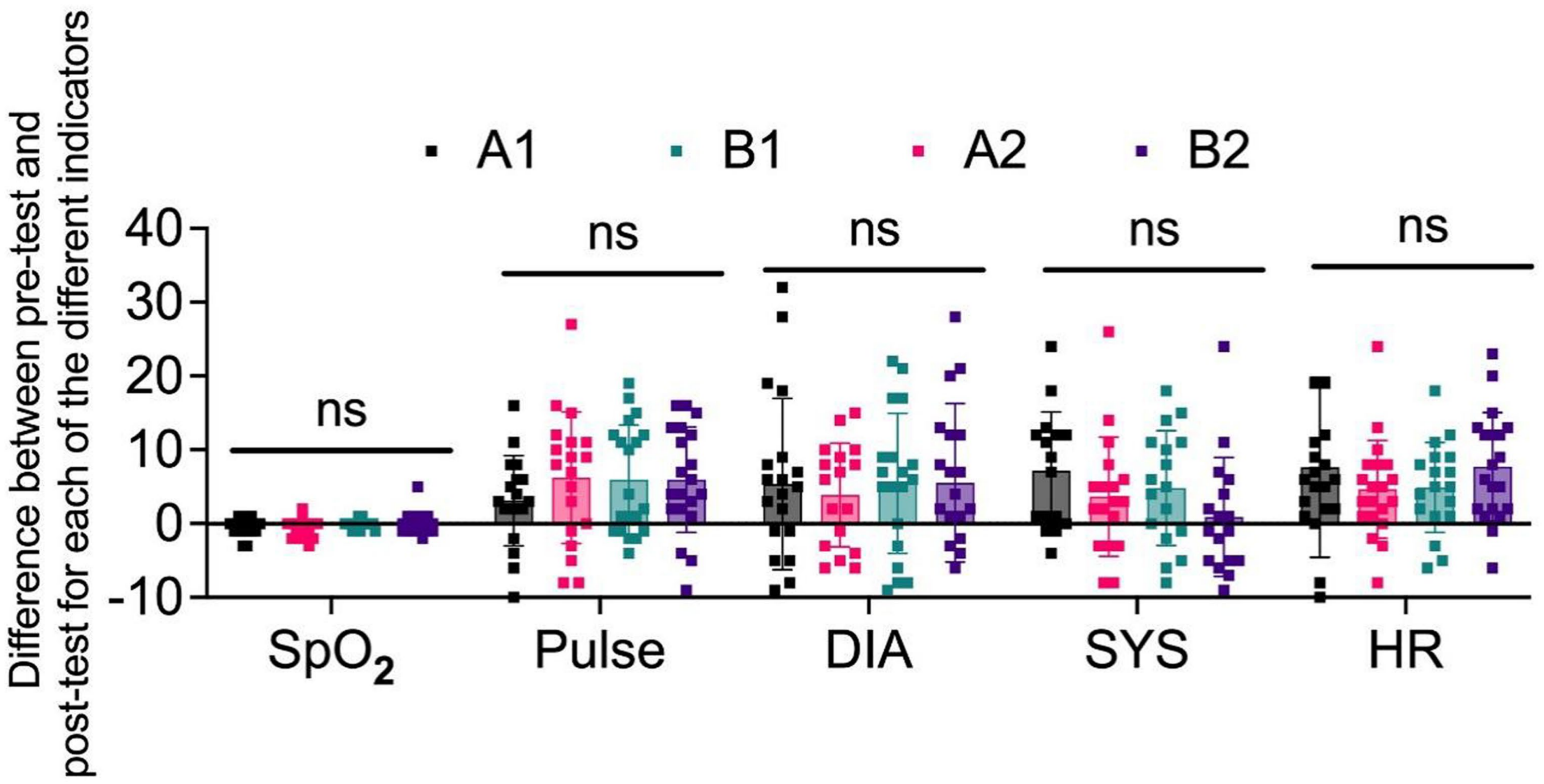
The difference between pre-test and post-test for each of the different physical indicators in four groups of participants.

Furthermore, the variations in physiological metrics observed before and after testing for each group were examined to assess individual changes. The difference was calculated by subtracting the post-test value from the pre-test value. The results indicated that the differences in recovery among the groups were not statistically significant, as shown in Figure 3, with increases in blood oxygen levels ranging from 0 to 1. Additionally, the reductions in other metrics, such as blood pressure and pulse, primarily ranged between 0 and 10, with a few notable exceptions (Figure 3). This outcome implied that the differences in physiological indicators before and post-test across the parks are minimal, suggesting that the physiological recovery effects in each park were comparable and demonstrated a certain level of physiological recovery efficacy. It was noteworthy that in terms of systolic blood pressure indicators, the recovery values of pocket parks even surpassed those of community parks. This indicates that there are no significant differences in the recovery values of various physiological indicators among these four parks.

### 3.2 Psychological indices

#### 3.2.1 PANAS and POMS

Multivariate analysis of variance (MANOVA) was employed to compare means, focusing on pre- and post-test outcomes of the PANAS and POMS for each park. Within groups, pre- and post-test comparisons assessed recovery values after engagement in specific parks. Recovery effects were then compared across groups, analyzing variations in outcomes among parks. *Post-hoc* tests further explored significant differences in MANOVA results, enhancing understanding of how specific parks impact emotional recovery.

##### 3.2.1.1 PANAS

In the context of positive emotions (Table 5), the pre-test average score for Group A1 was 2.72 (SD = 0.70), increasing to 2.87 (SD = 0.754) post-test, indicating improved positive emotions with a concentrated score distribution. Group A2’s pre-test average was 2.47 (SD = 0.76), rising to 3.11 (SD = 0.98) post-test, showing a more pronounced improvement in positive emotions alongside increased variability. Group B1 had a pre-test average of 2.87 (SD = 0.62) and a post-test average of 2.91 (SD = 0.89), reflecting slight improvement in positive emotions with notable score variability. Finally, Group B2’s pre-test average was 2.45 (SD = 0.65), increasing to 2.87 (SD = 0.72) post-test, also indicating improved positive emotions with a slight variability increase.

**Table 5:** (A) The mean value and SD of PANAS; (B) Testing the inter-subject effects of PANAS.

A									
Measures	Time	A1		A2		B1		B2	
		Mean	SD	Mean	SD	Mean	SD	Mean	SD
Positive	Pre	2.72	0.70	2.47	0.76	2.87	0.62	2.45	0.66
	Post	2.87	0.75	3.11	0.98	2.92	0.89	2.87	0.72
Negative	Pre	1.79	0.75	1.82	0.70	2.27	0.74	2.24	0.72
	Post	1.35	0.42	1.34	0.64	1.33	0.40	1.33	0.40

B					
Indicators		f	F	*p*	ηp^2^
Group	Positive	3	0.616	0.606	0.012
	Negative	3	1.742	0.161	0.033
Time	Positive	1	6.727	0.010	0.042
	Negative	1	50.878	<0.001	0.251
Group*	Positive	3	1.215	0.306	0.023
Time	Negative	3	1.952	0.124	0.037

In the context of negative emotions (Table 5), Group A1’s pre-test average was 1.79 (SD = 0.75), decreasing to 1.35 (SD = 0.42) post-test, indicating reduced scores and variability. Group A2’s pre-test average was 1.815 (SD = 0.70), dropping to 1.335 (SD = 0.64) post-test, reflecting a decrease in scores and a slight increase in variability. Group B1’s pre-test average was 2.27 (SD = 0.74), falling to 1.33 (SD = 0.39) post-test, showing a significant decline in scores and reduced variability. Finally, Group B2’s pre-test average was 2.24 (SD = 0.72), decreasing to 1.33 (SD = 0.39) post-test, also indicating a significant decrease in scores and reduced variability.

The findings of a two-way ANOVA, which assessed the between-subjects effects of the PANAS (Positive and Negative Affect Schedule) across the Group and Time factors, are detailed in Table 5. Between Groups, neither positive affect (*F* = 0.616, *p* = 0.606, ηp^2^ = 0.012) nor negative affect (*F* = 1.742, *p* = 0.161, ηp^2^ = 0.033) showed significant differences. Time effects were significant for both positive affect (*F* = 6.727, *p* = 0.010, ηp^2^ = 0.042) and negative affect (*F* = 50.878, *p* < 0.001, ηp^2^ = 0.251), with a particularly strong effect observed for negative affect. Interaction effects (Group × Time) were non-significant for both positive affect (*F* = 1.215, *p* = 0.306, ηp^2^ = 0.023) and negative affect (*F* = 1.952, *p* = 0.124, ηp^2^ = 0.037). Overall, Time significantly influenced both positive and negative affect, while Group effects and Group × Time interactions did not show significant differences.

##### 3.2.1.2 POMS

From Table 6, it could be observed that there were no significant effects or differences among the different groups regarding anxiety (*F* = 1.71, *p* > 0.05), anger (*F* = 0.632, *p* > 0.05), depression (*F* = 1.286, *p* > 0.05), vigor (*F* = 0.200, *p* > 0.05), fatigue (*F* = 0.317, *p* > 0.05), and confusion (*F* = 0.908, *p* > 0.05). However, the differences were significant when comparing the pre-test and post-test results within each group. *Post-hoc* tests reveal that, at the temporal level, anxiety (*F* = 45.219, *p* < 0.05), anger (*F* = 10.148, *p* < 0.05), depression (*F* = 28.891, *p* < 0.05), vigor (*F* = 47.484, *p* < 0.05), fatigue (*F* = 47.384, *p* < 0.05), and confusion (*F* = 55.315, *p* < 0.05) all show significant performance (Table 6). Furthermore, the interaction between group and time indicates no significant differences in psychological indicators, suggesting that the effects of group and time on these physiological parameters were independent, with no significant interaction effects.

**Table 6:** Results of multivariate analysis of variance (ANOVA) for psychological variables on the POMS, including degrees of freedom and F statistics.

	Tension	Anger	Depression	Vigor	Fatigue	**Confusion**
Group	1.71	0.632	1.286	0.200	0.317	0.908
Time	45.219***	10.148**	28.891***	47.484***	47.384***	55.315***
Group*Time	1.439	0.53	0.964	1.177	0.898	1.547

*p < 0.05; **p < 0.01; ***p < 0.001.

**Figure 4:**
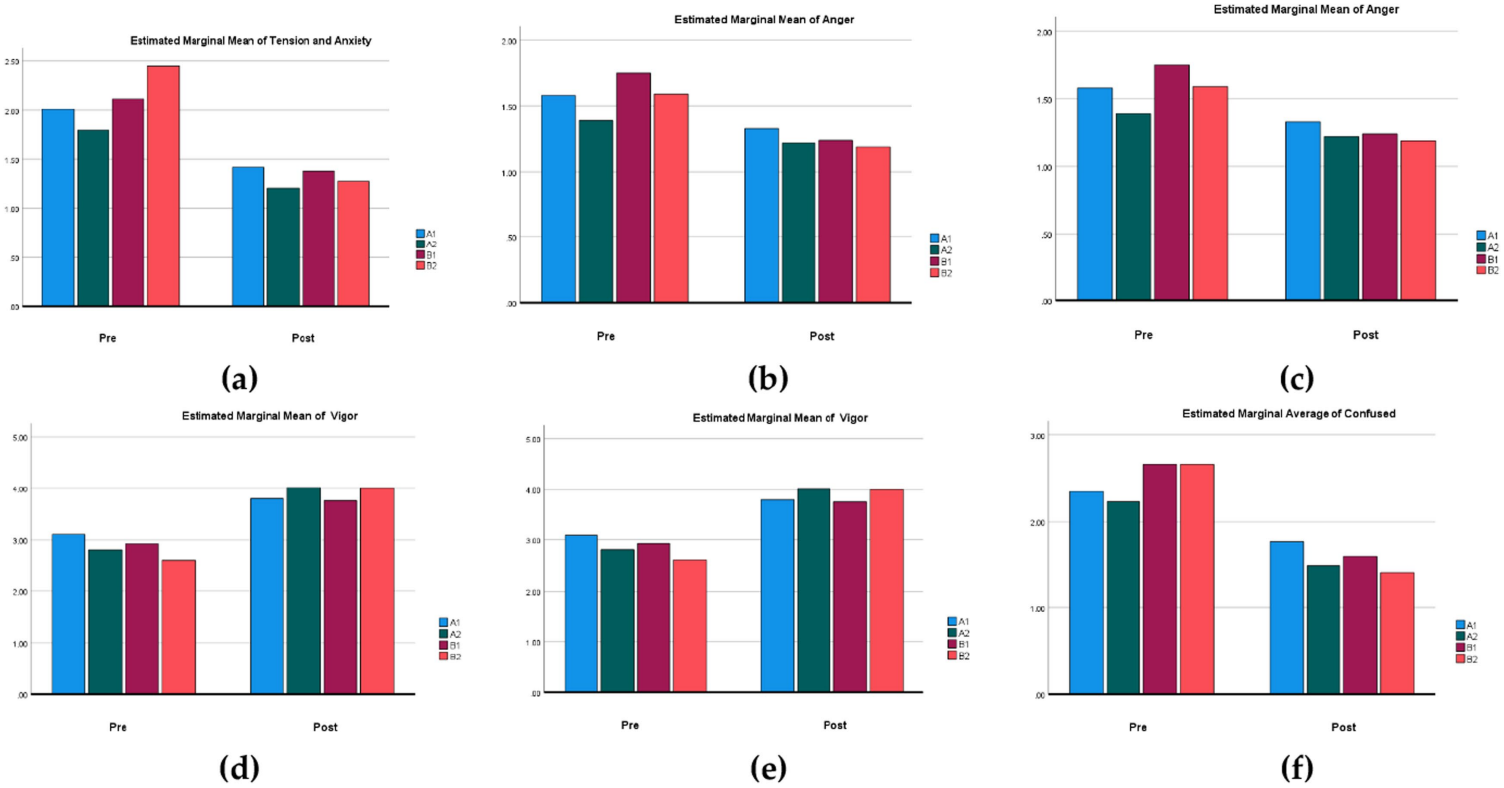
Comparison of mean differences before and after the POMS scale: **(a)** estimated marginal mean of tension and anxiety; **(b)** estimated marginal mean of anger; **(c)** estimated marginal mean of depression; **(d)** estimated marginal mean of vigor; **(e)** estimated marginal mean of fatigue; **(f)** estimated marginal mean of confuse.

From Figure 4, it was evident that the average values of various POMS indicators show a significant positive trend in both the pre-test and post-test across the four parks. This indicated that both pocket parks and community parks had a beneficial impact on psychological recovery, effectively reducing negative emotions and enhancing vitality.

#### 3.2.2 PRS and ROS

##### 3.2.2.1 PRS

Table 7 shows the I-J values and significance comparisons for each dimension of the PRS calculated through *post-hoc* variance analysis.

**Table 7:** Comparison of variance of PRS *post-hoc* tests in different parks.

Scale	Type	A1		A2	B1			B2	
		I-J	Sig.	I-J	Sig.	I-J	Sig.	I-J	Sig.
Being away	A1	—		−1.380***	0.001	−0.500	0.217	−1.090**	0.008
	A2			—		0.880^*^	0.032	0.290	0.473
	B1					—		0.590	0.146
	B2							—	
A Fascination	A1	—		−1.875***	0.001	−1.383*	0.013	−1.658**	0.003
	A2			—		0.491	0.370	0.216	0.692
	B1					—		−0.275	0.616
	B2							—	
Compatibility	A1	—		−0.275	0.296	−0.066	0.799	−0.108	0.680
	A2			—		0.208	0.428	0.166	0.526
	B1					—		−0.041	0.874
	B2							—	
Consistency	A1	—		−0.900*	0.018	−0.460	0.220	−0.800*	0.035
	A2			—		0.440	0.241	0.100	0.789
	B1					—		0.340	0.364
	B2							—	

*p < 0.05; **p < 0.01; ***p < 0.001.

In the “Being away” dimension, the I-J effect value for type A1 under condition A2 was −1.380, with a significance level of 0.001 (denoted as ***p < 0.001), indicating a significant difference between types A1 and A2. Similarly, the I-J effect value under condition B2 is −1.090, which also showed significance at a level of 0.008 (**p < 0.01). The I-J effect value for type A2 under condition B1 is 0.880, significant at 0.032 (*p < 0.05). This suggested significant differences in the restorative effects of different pocket parks in the “Being away” dimension, with pocket parks with a wide variety of plant species demonstrating stronger restorative qualities in this aspect.

In the “Fascination” dimension, the I-J effect value for type A1 under condition A2 was −1.875, which was extremely significant (***p < 0.001). The I-J effect value under condition B2 is −1.658, also showing significance at a level of 0.003 (**p < 0.01). The I-J effect value under condition B1 is also −0.275, but the significance level is low (*p* = 0.616). This indicated significant differences in the restorative effects of different pocket parks in the “Fascination” dimension, with those featuring a wide variety of plant species exhibiting stronger restorative qualities. However, there was no significant difference between community parks and pocket parks, suggesting that their restorative levels in this dimension are comparable.

In the “Compatibility” dimension, none of the I-J effect values reached statistical significance, indicating no significant differences between the types under these conditions. It suggested that the restorative values of pocket parks in terms of compatibility can reach the levels of community parks.

In the “Consistency” dimension, the I-J effect value for type A1 under condition A2 is −0.900, which was significant at a level of 0.018 (*p < 0.05), while the I-J effect value under condition B2 is −0.800, also showing significance at a level of 0.035 (*p < 0.05). This indicated that in the dimension of consistency, pocket parks primarily focused on activity space are slightly inferior to those primarily focused on plants and community parks.

##### 3.2.2.2 ROS

In the comparison of recovery outcomes (ROS) from Table 8, the six questions of the ROS scale were aggregated, and the final score for each individual was calculated by summing these responses. A significance analysis was then conducted on the differences observe. The findings revealed that there were no significant differences in ROS scores among the various groups, indicating that there is no notable difference in psychological recovery effects between different types of pocket parks and various types of community parks.

**Table 8:** Comparison of the variance of ROS *post-hoc* tests in different parks.

I	J	I-J	Sig.	95% confidence interval	
				Upper-bound	Lower-bound
A1	A2	−3.00	0.059	−6.11	0.11
	B1	−0.85	0.588	−3.96	2.26
	B2	−2.55	0.107	−5.66	0.56
A2	A1	3.00	0.059	−0.11	6.11
	B1	2.15	0.173	−0.96	5.26
	B2	0.45	0.774	−2.66	3.56
B1	A1	0.85	0.588	−2.26	3.96
	A2	−2.15	0.173	−5.26	0.96
	B1	−1.70	0.280	−4.81	1.41
B2	A1	2.55	0.107	−0.56	5.66
	A2	−0.45	0.774	−3.56	2.66
	B1	1.70	0.280	−1.41	4.81

### 3.3 Assessment of the relationship between park elements and restoration effects of pocket park and community park

#### 3.3.1 Subjective evaluation system for park element indicators for physiological/psychological restoration

**Figure 5:**
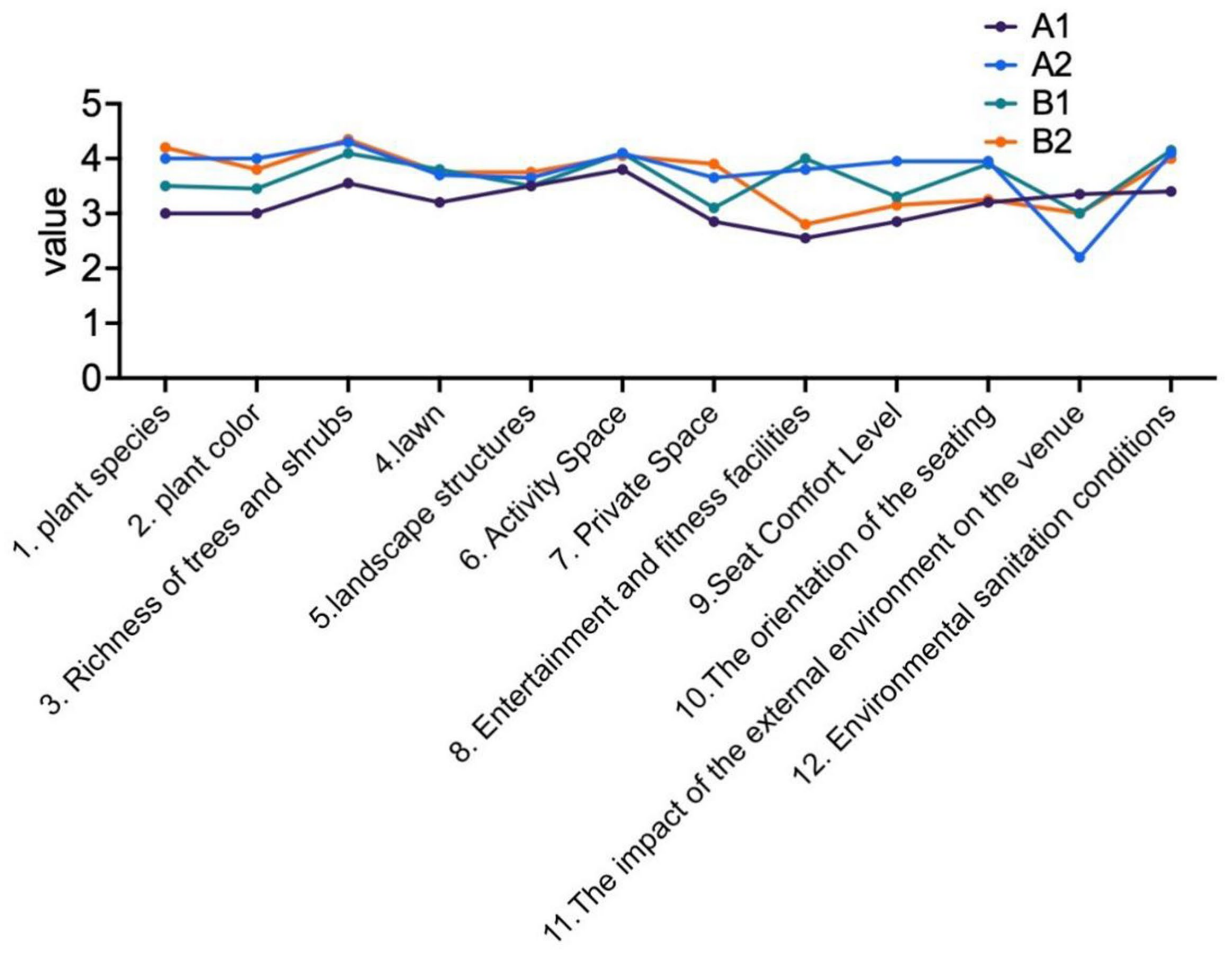
The average subjective ratings of different environmental elements across four parks.

During the experimental process, each participant was asked to complete a scale measuring environmental factors within the park, rating the restorative effects of various environmental elements. Ultimately, the average scores for the vegetation across the four parks were calculated, as illustrated in Figure 5. In the scoring calculation, Question 11 addressed the minimal impact of external environmental factors on your visiting experience, which was reverse-scored to reflect the influence of external conditions. Analyzing the scores for each environmental factor reveals that the parks in Groups A1 and B1, which predominantly feature artificial elements, received lower ratings in the vegetation category. In contrast, the pocket park in Group A2 was rated positively regarding overall environmental factors (Figure 5).

This research investigates the potentially significant relationship between Restoration Outcomes and Perceived Restoration and the assessment of environmental variables within the venue, employing Pearson correlation analysis via SPSS 27.0.

**Figure 6:**
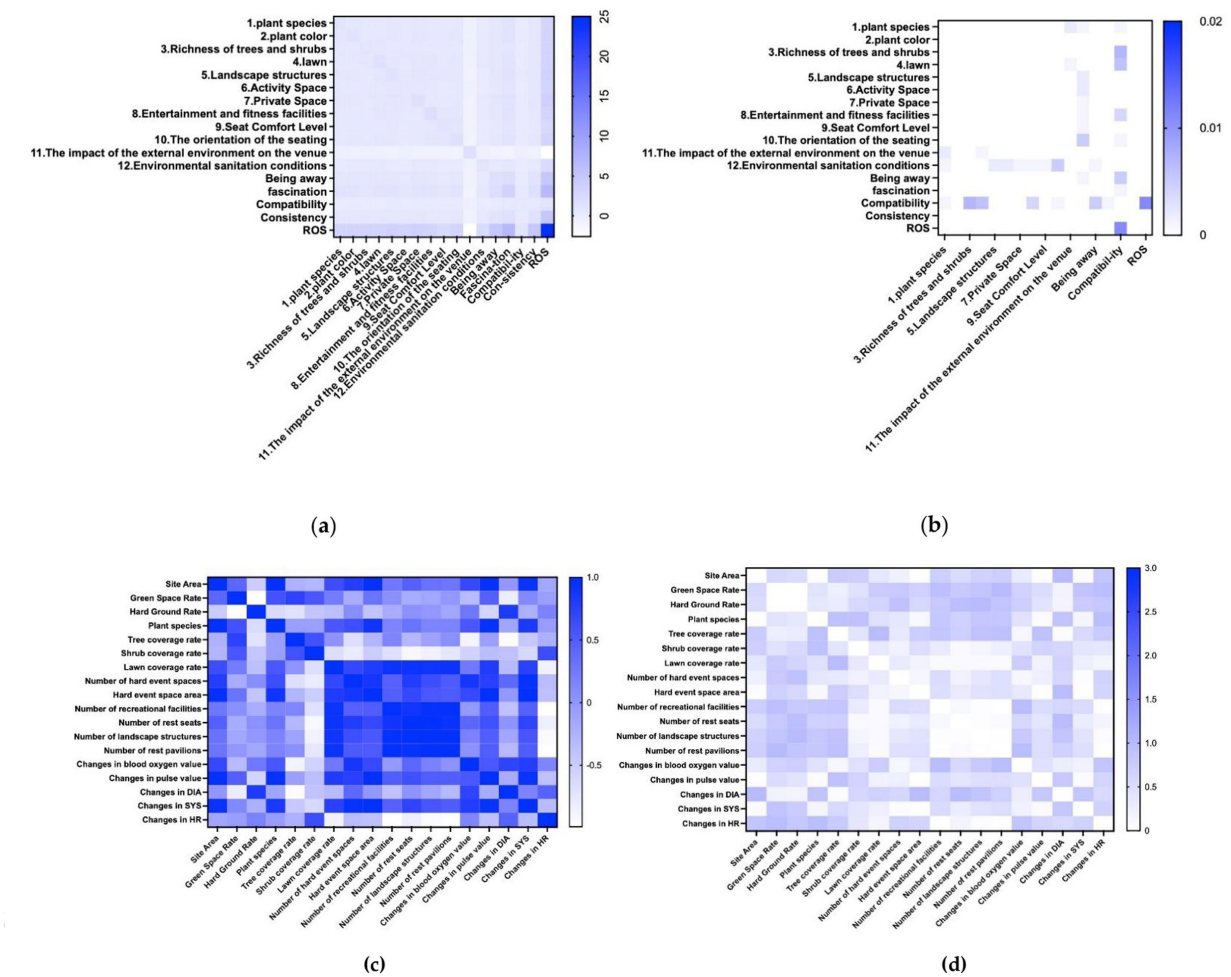
The correlation analysis: **(a)** analysis of covariance; **(b)** significance analysis; **(c)** analysis of Pearson correlation; **(d)** significance analysis.

Through bivariate correlation analysis, it could be observed that the correlation analysis indicated a strong relationship between the four dimensions of PRS and the overall value of ROS with the subjective evaluation scores of various landscape element indicators, allowing for regression analysis to be conducted. Figure 6 illustrates the covariance and significance among multiple indicators. Most indicators show a positive correlation with clear significance, while the impact of the external environment is negatively correlated with the other values (Figure 6).

A multiple linear regression equation was established to assess the relationship between park elements and psychological restoration (ROS) by rating element indicators of pocket and community parks. Model 1 shows an unstandardized coefficient (B) for the constant term of 7.845 (SE = 1.819, *t* = 4.314, Sig. < 0.001), confirming its significance. The coefficient for activity space is 3.777 (SE = 0.442, Beta = 0.695, *t* = 8.546, Sig. < 0.001), indicating a significant positive effect on total ROS. The variance inflation factor (VIF) is 1.000, indicating no multicollinearity issues. Model 2 adds richness of trees and shrubs as predictors, decreasing the constant term to 6.087 (SE = 1.840, *t* = 3.308, Sig. = 0.001). The coefficient for activity space is 2.732 (Beta = 0.503, *t* = 4.926, Sig. < 0.001), still showing a significant effect. The richness of trees and shrubs had a coefficient of 1.460 (SE = 0.503, Beta = 0.297, *t* = 2.904, Sig. = 0.005), indicating their positive impact on total ROS (Table 9). This finding indicated that activity space significantly impacts the overall recovery value of ROS in relation to tree and shrub richness, resulting in the following regression equation (Equation 1).


(1) y=6.087+0.503x1+0.297x2

**Table 9:** (a) Linear-regression analysis of restoration outcomes and the physical environment of the four parks; (b) Linear regression analysis of SYS and the physical environment of the four parks.

A								
Coefficient								
Model		Non-standardized coefficient		Standardized coefficient	*t*	Sig.	Collinearity	
		B	Standard error	Beta			Tolerance	VIF
1	(constant).	7.845	1.819		4.314	<0.001		
	6. Activity space	3.777	0.442	0.695	8.546	<0.001	1.000	1.000
2	(constant).	6.087	1.840		3.308	0.001		
	6. Activity space	2.732	0.555	0.503	4.926	<0.001	0.580	1.725
	3. Richness of trees and shrubs	1.460	0.503	0.297	2.904	0.005	0.580	1.725
Dependent variable: Total ROS								

Model abstract								
				Changed value				
	R	R^2^	Adjust R^2^	Change in R^2^ value	Change in *F* value	f1	f2	Change in Sig. value
1	0.695a	0.484	0.477	0.484	73.037	1	78	0.001
2	0.731b	0.535	0.522	0.051	8.432	1	77	0.005
Predictor variables: (constant), 6. Activity space Predictor variables: (constant), 6. Activity space, 3. Trees and shrubs Dependent variable: Total ROS								

B								
Coefficient								
Model		Non-standardized coefficient		Standardized coefficient	*t*	Sig.	Collinearity	
		B	Standard error	Beta			Tolerance	VIF
1	(constant).	−0.771	0.579		−1.330	0.315		
	Hard event space area	0.001	0.000	0.981	7.065	0.019	1.00	1.000
b. Dependent variable: change in SYS (Systolic blood pressure)								

The results demonstrated that the quality of activity space and the richness of trees and shrubs significantly influence overall restoration value. The following regression equation was derived, where y represents the recovery effect of ROS, and x1 and x2 represent the activity space and the quality of trees and shrubs, respectively. The equation indicated that in the context of pocket parks and community park environments, for each additional unit of active space, the restorative effect increased by 0.503 units; similarly, for each additional unit of trees and shrubs, the restorative effect increased by 0.297 units.

#### 3.3.2 Objective evaluation system for park element indicators for physiological

When analyzing the correlation between objective spatial indicators and variations in physiological metrics of youth (measured as the difference between pre- and post-test values), a strong positive correlation emerges between factors such as space area, vegetation types, lawn coverage, and hardscape elements with changes in pulse and systolic blood pressure. Additionally, the proportion of hard surfaces showed a significant positive correlation with diastolic blood pressure changes. Conversely, a significant negative correlation was found between heart rate variability and the quantity of recreational facilities and landscape structures, whereas the link between active spaces and blood oxygen levels was comparatively weak (Figure 6).

Model 1 reflected the impact of hard space area on the variation of SYS. The constant term (B = −0.771, SE = 0.579) is insignificant (*t* = −1.330, Sig. = 0.315), indicating no significant baseline SYS variation when controlling for hard space area. The unstandardized coefficient for hard space area was 0.001 (SE = 0.000), with a standardized coefficient (Beta) of 0.981 (*t* = 7.065, Sig. = 0.019), indicating a significant positive correlation; as hard space area increases, SYS variation also increased. Tolerance and VIF for this variable were both 1.000, showing no multicollinearity (Table 9). The model’s R-value was 0.981, indicating a strong linear relationship, and the R-squared (R^2^) value of 0.961 means the model explains 96.1% of SYS variation, demonstrating high explanatory power. From the above analysis, a standardized model was derived in Equation 2.


(2) y=−0.771+0.981x

In this equation, y represented the change in systolic pressure, while x represents the area of hard activity space, indicating that for each unit increase in hard activity space, systolic pressure decreases by 0.981 units. Hard activity spaces can effectively reduce Systolic pressure.

## 4 Discussion

The results show that a 20-min visit to a pocket park induces physiological and psychological recovery comparable to that of a community park. Both park types positively impact various physiological and psychological metrics. This supports the initial hypothesis that well-designed pocket parks can offer restorative benefits similar to community parks. It also aligns with previous research, demonstrating that small urban green spaces can reduce psychological stress, enhance positive emotions, and promote physiological health (15–17, 29). However, differences in recovery outcomes indicate that pocket and community parks produce varying effects across physiological and psychological measures.

### 4.1 Physiological restoration

Although all four parks showed positive trends in physiological indicators, pocket parks lagged slightly behind community parks in pulse, heart rate, and diastolic pressure. This may be due to differences in park size, activity range, and amenities. Community parks, with their larger area and diverse facilities, offer greater opportunities for exercise and recreation. Features like walking trails, fitness equipment, and playgrounds encourage physical activity, which has been linked to lower blood pressure (39, 40). Short walking sessions are particularly effective (32), and the ample space in community parks facilitates such activities, promoting overall health. While pocket parks also include fitness facilities, their smaller size limits exercise opportunities. However, they can still enhance physiological recovery by integrating walking paths, exercise stations, and shaded seating to increase use. A welcoming atmosphere fosters social interaction and community engagement, which is essential for mental health.

Correlation analysis revealed a strong positive relationship between hard activity space and blood pressure changes. This suggests indirect effects, as walking and recreational activities in these spaces can improve psychological well-being, influencing physiological recovery. While previous studies emphasize the restorative effects of natural elements like vegetation and water features, this research highlights the significant role of artificial spaces. Hardscapes, such as plazas, can provide attentional and emotional benefits comparable to forests, waterfronts, and grassy areas (41). These psychological benefits may, in turn, support physiological recovery. To maximize these effects, communities and campuses with high youth populations should incorporate more interactive activity spaces. Enhancing social engagement and recreation in these environments can promote both physical and mental well-being.

Furthermore, blood oxygen recovery across the four parks is not significant but shows some improvement, indicating that both pocket and community parks, despite their size, contribute to increased blood oxygen levels. Recovery values among the parks remain relatively similar. Heart rate variation reveals a negative correlation with recreational facilities and landscape structures but a positive correlation with vegetation. Physical activity naturally increases heart rate, with intensity directly affecting the magnitude of the rise (42). In contrast, vegetated environments promote tranquility, helping lower the heart rate. This underscores the complex relationship between surroundings and physiological responses, warranting further research.

### 4.2 Psychological restoration

#### 4.2.1 PANAS and POMS

This study confirms that short-term exposure to urban natural environments enhances positive emotions and reduces negative emotions in young individuals. Previous research highlights the benefits of natural settings, such as forests, for emotional well-being (43, 44). Additionally, studies suggest that walking in urban suburbs provides emotional restoration comparable to forest environments (32). This study further demonstrates that small green spaces in high-density urban areas offer significant psychological benefits, with pocket parks achieving restorative effects similar to community parks. These findings suggest that even brief exposure to green spaces can improve emotional health, particularly in urban environments where individuals feel disconnected from nature.

Short-term exposure to natural environments significantly boosts positive emotions and reduces negative ones, particularly in densely urban areas. This effect may stem from nature’s restorative qualities, offering relief from urban stress. For example, admiring colorful plants like flowers and vibrant leaves evokes joy, while strolling through green spaces promotes physical activity, which enhances vitality and alleviates negative emotions. Additionally, using recreational and fitness facilities in parks can further boost happiness.

#### 4.2.2 PRS and ROS

In evaluating “Being away,” notable restorative differences emerged between Pocket Parks A1 and A2, as well as distance disparities with Community Park A2. However, no significant differences in restorative effects were found between Pocket Park A1 and Community Park B1, underscoring the crucial role of vegetation. Notably, Pocket Park A2 surpasses Community Park B1 in restorative value due to its diverse plant communities, which enhance sensory experiences. In contrast, Community Park B1, despite its larger space, has limited plant coverage, potentially reducing its restorative impact. Additionally, its proximity to urban noise undermines perceived tranquility, complicating comparisons. These findings highlight the significance of distance and natural environment quality in restorative experiences, aligning with prior research on vegetative environments and the “away” concept. Peng et al. further emphasize that plant species diversity and greenery proportion are key factors in assessing a park’s restorative potential in the “away” dimension (30).

Regarding “fascination,” significant differences were observed between Pocket Park A1 and the other three parks. This may stem from A1’s smaller size, limited plant variety, and lack of facilities. In contrast, Pocket Park A2, with its greater plant diversity and enhanced recreational amenities, showed smaller differences in fascination compared to the two community parks. This suggests a positive correlation between landscape richness and restorative outcomes.

In terms of “compatibility,” no significant differences emerged among the four parks, likely due to their generally favorable environmental conditions and complementary interactions among elements. Finally, regarding “consistency,” parks with greater vegetation emphasis demonstrated superior restorative effects, indicating that plant environments play a decisive role in this dimension.

An examination of the Recovery Outcome Scale (ROS) found no significant overall differences in restorative effects among the four parks. This suggests that pocket parks offer psychological restoration comparable to community parks, enhancing vitality and reducing stress. However, I-J value comparisons revealed that Pocket Park A2 had a superior restorative effect compared to A1 and even slightly outperformed the two community parks. This may be due to its well-balanced design, featuring rich plant space, ample activity areas, and well-organized recreational facilities.

Additionally, regression analysis linking park restorative effects with park element evaluations showed a strong correlation between restoration and both plant space and hard activity space. While pocket and community parks provide similar restorative benefits overall, Pocket Park A2’s distinct attributes make it particularly effective for psychological restoration, warranting further exploration of its successful design elements.

### 4.3 The relationship between green space quality and public well-being

Our findings reveal that pocket parks and community parks, distinguished by diverse park elements, sufficient vegetation cover, and comprehensive facilities, exhibit significant restorative effects on both physical and mental well-being. This suggests a positive correlation between urban green space quality and public welfare, particularly in high-density urban environments. The implementation of accessible, high-quality green spaces through initiatives such as “greening the gaps” provides restorative benefits to residents’ health, a conclusion supported by prior research (45, 46). These results underscore the importance of biophilic design principles (47). Research further highlights the critical role of green infrastructure in mitigating the urban heat island effect, enhancing residential comfort and promoting residents’ well-being (48). Future studies should incorporate these factors as mediating variables.

However, in high-density urban environments, noise pollution constitutes a critical factor influencing residents’ psychological well-being. While the noise mitigation capacity of small green spaces may not match that of larger green areas, we can still attenuate noise levels by enhancing the quality of these spaces. For instance, strategic tree planting around small parks can effectively reduce noise, create more private spaces, and provide residents with a more immersive green experience.

### 4.4 The limitations and future research directions

The experiment’s results may be influenced by subjective factors and variations in participants’ physical fitness, potentially introducing bias. Additionally, individual differences in esthetic preferences and perception should be considered (49). For example, some students may prefer green spaces, while others favor sports activities, leading to varying recovery outcomes. These differences in esthetic preference among young people contribute to variations in restoration effects, warranting further study. Concerning this study focused exclusively on young adults to enhance feasibility and practicability rather than to represent diverse age demographics. Future research should investigate generalizability of the findings and esthetic preferences across border demographic groups (e.g., older adults, children).

This article examines the restorative effects of pocket and community parks on young individuals through physiological and psychological assessments, identifying key park elements that aid recovery. However, landscape restoration is rarely driven by a single factor, such as vegetation or activity spaces. Instead, multiple landscape features interact to form cohesive restorative environments. The complex relationship between green spaces and well-being underscores the value of even small, well-designed parks as vital urban sanctuaries. Social interactions within these spaces also play a crucial role, fostering connections that enhance the restorative experience. A holistic approach to park design and evaluation is essential to maximize their benefits in urban landscapes.

The absence of social interaction in this study may compromise its ecological validity (50). However, this research prioritizes an examination of the differential restorative effects of pocket parks versus community parks, rather than an exploratory analysis of the impact of natural environments on psychological restoration. The former emphasizes internal validity, while the latter prioritizes ecological validity. Future research should, therefore, conduct a more in-depth analysis at the level of ecological validity to facilitate broader application within natural settings.

### 4.5 Policy implication*s*

It is evident from the aforementioned research that pocket parks and community parks can achieve complementary effects on human psychological and physiological restoration, and parks with high environmental quality can achieve better restorative effects even if they are small in size. Therefore, in highly urbanized contexts, the presence of micro-scale green spaces correlates with improved physiological health and psychological restoration among residents. This observation highlights the necessity of a strategic approach to integrating green spaces in proximity to residential areas within high-density urban settings. Such pocket parks should be strategically designed, incorporating multiple access points and structured community initiatives to maximize their utility for the local populace. In high-density urban environments, the strategic deployment of multiple small-scale green spaces can improve park resource accessibility in central areas and socioeconomically disadvantaged neighborhoods (51–53). This strategy mitigates the constraints experienced by residents who may have limited time for travel to larger green spaces for restorative experiences (54). Moreover, the incorporation of interactive and activity-based zones in park layouts, alongside the provision of recreational and fitness facilities, can effectively address the activity requirements of inhabitants, thereby promoting both physical health and psychological wellness.

## 5 Conclusion

This study utilized field experiments to evaluate the restorative effects of pocket parks and community parks with varying green space proportions. A pre-test and post-test methodology evaluated physiological and psychological indicators in youth to compare outcomes across park types. A comprehensive evaluation system was developed to assess restorative differences among park elements, integrating subjective and objective indicators. A preliminary field survey quantitatively analyzed environmental factors at various sites, leading to the formulation of a park environment restoration assessment questionnaire. A regression analysis was conducted, incorporating objective indicators, physiological recovery data, and environmental assessment questionnaires related to psychological recovery scales. Results revealed that pocket parks with rich landscape elements can achieve restorative effects comparable to larger community parks. Physiologically, activity space significantly influenced blood pressure changes in youth. Psychologically, different elements impacted various indicators; both activity space and plant environments similarly enhanced vitality and positive emotions and reduced negative emotions, while the plant environment had a more pronounced effect on stress relief. This suggests that elements exert varying degrees of restorative effects on physiological and psychological indicators, either independently or synergistically enhancing overall well-being. Therefore, park design should prioritize the “quality” of the environment, enriching landscape elements and maintaining surrounding facilities. In densely populated urban areas, there is a pressing need for the development of more “small yet exquisite” parks.

## Data Availability

The raw data supporting the conclusions of this article will be made available by the authors, without undue reservation.
